# Replicating outdoor environments using VR and ambisonics: a methodology for accurate audio-visual recording, processing and reproduction

**DOI:** 10.1007/s10055-024-01003-1

**Published:** 2024-05-17

**Authors:** Fotis Georgiou, Claudia Kawai, Beat Schäffer, Reto Pieren

**Affiliations:** Empa, Swiss Federal Laboratories for Materials Science and Technology, Laboratory for Acoustics/Noise Control, Überlandstrasse 129, Dübendorf, 8600 Switzerland

**Keywords:** Virtual reality, Ambisonics, Decoder equalisation, Perceptual evaluation

## Abstract

This paper introduces a methodology tailored to capture, post-process, and replicate audio-visual data of outdoor environments (urban or natural) for VR experiments carried out within a controlled laboratory environment. The methodology consists of 360^∘^ video and higher order ambisonic (HOA) field recordings and subsequent calibrated spatial sound reproduction with a spherical loudspeaker array and video played back via a head-mounted display using a game engine and a graphical user interface for a perceptual experimental questionnaire. Attention was given to the equalisation and calibration of the ambisonic microphone and to the design of different ambisonic decoders. A listening experiment was conducted to evaluate four different decoders (one 2D first-order ambisonic decoder and three 3D third-order decoders) by asking participants to rate the relative (perceived) realism of recorded outdoor soundscapes reproduced with these decoders. The results showed that the third-order decoders were ranked as more realistic.

## Introduction

Urbanisation with continuously increasing population and mobility results in increasing noise pollution and a decline of green spaces, although the latter are important for restoration from stress. To support the urban planning there is a need for studies on noise-related stress in combination with the restorative effects of green spaces. Besides field surveys, highly controlled laboratory VR experiments are a promising method to study, quantify and separate the different influencing factors.

The great advantage of using VR to investigate the effect of different environments on perception and/or health outcomes is that the measurements can be done under controlled conditions. One can, for instance, control the sound levels and the visual information and systematically change them to investigate their effects on stress. The challenge is to reproduce all the audio-visual information as accurately (i.e., physically correct and plausible to listen to) as possible in order to achieve a fully immersive experience.

The main focus of this paper is to present the methodology used to record audio-visual information of different urban outdoor environments and to reproduce them accurately in the laboratory. Special attention is given to the spatial audio reproduction, which was realised using ambisonics and reproduced via a spherical loudspeaker array. Ambisonics (see ambisonics theory in Sect. 2.2) has gained significant popularity in recent studies within the field of soundscape and VR (Guastavino et al. 2005; Davies et al. 2014; Vogt 2015; Shivappa et al. 2016; Kern and Ellermeier 2020; Robotham et al. 2022; Tarlao et al. 2022). In this paper, we evaluated in a listening experiment four ambisonic decoders (see Sect. 5.2) and developed equalisation methods both for the ambisonic recordings (see Sect. 4) and the decoders (see Sect. 5.3) aimed at achieving a high accuracy and plausibility in audio reproduction of outdoor soundscapes.

The four decoders which were evaluated through a listening experiment are the following: a 2D first-order ambisonics (FOA) decoder with max-${\textbf{r}}_{\text {E}}$ weights, a 3D third-order All-round ambisonic decoding (ALLRAD) decoder with max-${\textbf{r}}_{\text {E}}$ weights using a hemispherical loudspeaker set-up, the same decoder with an extended loudspeaker array including speakers below ear height, and a dual-band decoder.

The equalisation of ambisonic recordings and of the ambisonic decoders have been underrepresented in the literature so far. The decoder equalisation method presented here has similarities with a method developed for binaural reproduction of ambisonics via headphones (McKenzie et al. 2018). Regarding the method used for the equalisation of the ambisonic recordings, we are not aware of any previous published study applying it, although it might be important to consider it for accurate reproduction.

The work presented in this paper is part of the interdisciplinary Swiss research project RESTORE (Restorative potential of green spaces in noise-polluted environments; www.restore-project.ch), which aims at investigating the build-up of noise-related stress in combination with the restorative effects of green spaces and urban environments. One part of RESTORE contains experiments that use the controlled VR environment presented here to systematically investigate the build-up of and the restoration from short-term stress via skin conductance and salivary cortisol measurements.

The paper is organised as follows. The ’Theoretical background’ (Sect. 2), where we clarify the theoretical foundations that support the audio-visual recreation of outdoor environments, including the integration of ambisonic loudspeaker reproduction for audio and the utilisation of 360^∘^ video for visuals. ’Methodological overview’ (Sect. 3) provides an overview of the research methodology. ’Ambisonic recordings and equalisation’ (Sect. 4) examines the details of the ambisonic microphone used and the equalisation processes applied to refine the ambisonic recordings. Moving on to ’Ambisonic decoder variants’ (Sect. 5), we assess the performance of four ambisonic decoders employed in outdoor soundscape reproduction. Additionally, we detail the systematic methodology employed for decoder equalisation and calibration. Finally, ’360^∘^ audio-visual recording and playback’ (Sect. 6) discusses the audio and video recording setup used for data collection, which involved a 360^∘^ camera and a HOA microphone as well as the VR environment implemented in Unity for playback and user interaction.

## Theoretical background

### Loudspeaker vs headphone reproduction

Both loudspeaker (LS) and binaural reproduction via headphones have advantages and disadvantages (Brimijoin et al. 2013; Favrot 2010). The headphone reproduction is independent of the type of room and its acoustic properties, like the reverberation, sound reflections and background noise if using closed-back headphones. On the contrary, with loudspeaker reproduction, room treatment is required to minimise the effects of reflections on the sound-field and the effect of background noise. The performance of loudspeaker systems is influenced by the listener’s position. The ideal sound reproduction is perceived when the listener occupies the ’sweet spot,’ which is the position offering optimal audio quality and balance. In contrast, headphone listening does not involve considerations related to the sweet spot. In headphone reproduction, any audio sources are incorporated within the reproduction algorithm, so there is no need to handle loudspeaker positioning and delay compensation. Headphone systems are much cheaper compared to loudspeaker systems. For an ideal reproduction, the headphone system has to be tailored to each listener individually by incorporating their personalised head related transfer functions (HRTFs), something that is not required in loudspeaker reproduction. HRTFs are the frequency-dependent filters that capture how sound is altered as it travels from a sound source to a listener’s eardrums, taking into account the unique shape and characteristics of the listener’s head and ears. This is a time consuming process and on most occasions impossible to measure. Generic HRTFs can also be used, but source localisation is degraded and they increase the chances for front-back confusions and in-head localisation (these issues can be improved with the introduction of head-tracking but will not completely resolved). Besides these localisation issues in headphones, it is difficult to quantify the calibration uncertainty due to interpersonal differences in the HRTFs. With loudspeaker reproduction, sound sources are externalised and head tracking is not required, increasing the degree of realism. Playback level in headphone reproduction can only be measured using a head and torso simulator (HATS), a device designed to mimic the acoustic properties of a human head and torso, equipped with microphones in its ears, and not with a calibrated measurement microphone which records the sound pressure level. Finally, headphones are intrusive, mechanically from their weight, cables or contact pressure, and thermally by possible warming up of the ears.

Due to the above overview and argumentation, it was decided to use loudspeaker reproduction for the planned experiments. This is supported by recent work from Hong et al. (2019) on the quality assessment of acoustic environment reproduction methods for VR in soundscape applications, which showed that first-order ambisonics (FOA) reproduction via a 2D loudspeaker array (see ambisonics theory in Sect. 2.2) was rated overall as a better reproduction method compared to the FOA via headphones.

### Ambisonics

Gerzon (1975) introduced the theory of ambisonics. Ambisonics provides solutions for spatial audio recording and reproduction. The ambisonics theory can be split into two consecutive processes: firstly, the recording and encoding of the sound field and secondly, the accurate reconstruction of the sound field by decoding it to a speaker setup. Both of these processes have been extensively studied (Scaini and Arteaga 2014; Zotter et al. 2010; Zotter and Frank 2012; Zotter et al. 2012, 2015; Favrot 2010; Politis 2016; Politis and Gamper 2017). A good overview of the ambisonics can be found in Zotter and Frank (2019). The principle of ambisonics is to describe a sound field by spherical harmonic functions. Spherical harmonics are a hierarchical set of basis functions that are orthogonal upon the surface of a sphere. The ambisonic channels are denoted by degree *l* and order *m* of the spherical harmonic. The order of the ambisonic system describes the highest degree of the ambisonics channel in the signal set. The $0^{\text {th}}$-order ambisonics is simply one channel and represents the (scaled) omnidirectional sound pressure component of the sound field. In 3D first-order ambisonics (FOA), three extra figure of eight sound pressure components are added (the three spherical harmonics of degree 1) which represent the multipole expansion of the sound field surrounding the position of the listener. Higher-order ambisonics (HOA) consist of higher spherical harmonic degree channels, providing a more accurate representation of the sound field’s directional components. Compared to FOA, HOA is very attractive but has two main drawbacks: 1) It is costly since, for example, to capture 3D ambisonics of order $N = 4$ requires a microphone array with a minimum of $(N+1)^2 = 25$ channels/capsules and a loudspeaker layout to playback the 4^th^-order sound field, consisting also of at least $L = 25$ uniformly distributed loudspeakers covering the sphere; 2) Processing and reproduction are more challenging.

Ambisonics has some properties which make it more attractive compared to other loudspeaker reproduction techniques such as Wave Field Synthesis (Zotter and Frank 2012; Zotter et al. 2015; Favrot 2010). These are the following: (1) There is an associated recording technology, which is not the case with Wave Field Synthesis; (2) As opposed to channel-based formats, the stored channels are decoupled from the reproduction channels. Encoding is independent of decoding, meaning that you can encode 3^rd^-order ambisonics, but if the speaker layout cannot support it, you can decode it to a lower-order; (3) It can provide a smooth listening experience from all directions; (4) Since both recording and reproduction are defined on a sphere, the sound field can be rotated. However, it has also some disadvantages, which have pushed back ambisonics from becoming a more commonly used spatial audio method Zotter et al. (2015); Scaini and Arteaga (2014). Firstly, the sweet spot is small. Secondly, perceptual artifacts such as coloration, which is the alteration or distortion of the sound signal that adds unwanted artifacts to the audio playback can occur due to the fact that the reproduction is implemented by coherent loudspeaker signals. Thirdly, the performance of low-order ambisonics is considered poor, which can be handled by increasing the order; but, as previously mentioned, this will also increase the costs and the complexity of the processing.

Ambisonic recordings are taken using spherical microphone arrays. There are various first-order spherical microphone arrays available on the market such as the SoundField SPS200 microphone and the RØDE NT-SF1 but only few higher-order ones such as the Zylia ZM-1 (3^rd^-order) and the mhacoustics Eigenmike EM32 (4^th^-order). The signals captured via these microphone arrays cannot be used directly for playback. They must first be processed in the spherical harmonics domain (encoding) to produce the B-Format signals. The main challenge at this stage is the design of the radial filters to compensate for the radial structure of the sound field captured with the microphone, which basically scale the amplification gain of the spherical harmonics modes. The goals of these radial filters is to achieve a balance between the recovery of the sound field coefficients while keeping the noise amplification in the encoded signals at acceptable levels (Politis 2016; Politis and Gamper 2017). Companies such as SoundField, Zylia and mhacoustics provide their own software for encoding the signals captured from their microphones. There are also toolboxes and plug-ins available such as the SPARTA suite developed by Aalto University (Mccormack and Politis 2019) that allow the users to experiment with different encoding methods and decide which one suits their needs the best.

Decoding of the ambisonic signals ${\textbf{x}}$ into loudspeaker feeds ${\textbf{s}}$ is implemented using the decoding matrix ${\textbf{D}}$ as follows:

1 $$\begin{aligned} {\textbf{s}} = {\textbf{D}} {\textbf{x}} \end{aligned}$$

where ${\textbf{s}} = [{\textbf{s}}_1, {\textbf{s}}_2... {\textbf{s}}_L]^T$, ${\textbf{D}}$ is an $L \times (N+1)^2$ matrix and ${\textbf{x}} = [{\textbf{x}}_1, {\textbf{x}}_2,... {\textbf{x}}_{(N+1)^2 }]^T$. The goal of the decoder is to achieve the best possible reproduction for the specified loudspeaker layout (Zotter et al. 2015). Various decoding techniques have been developed. Two traditional decoding methods are the sampling decoding and the mode matching decoding. These methods are straightforward to implement but are not robust when the speaker setup is irregular or incomplete, which is the case in most real speaker layouts. Recent decoding methods are the All-round ambisonic decoding (ALLRAD) and the Energy-preserving decoding (EPAD) (Zotter and Frank 2012; Zotter et al. 2012). ALLRAD uses a combination of ambisonic decoding and vector-base amplitude panning (VBAP) developed by Pulkki (1997). First, it decodes the ambisonics signals to an optimal virtual loudspeaker layout (spherical t-designs Hardin and Sloane (1996)) and then uses VBAP to map the virtual loudspeaker signals to the real speakers. That property of ALLRAD gives this method a lot of flexibility. EPAD’s main goal is to achieve constant energy across all directions. For incomplete spherical loudspeaker layouts, EPAD is based on the design of a new set of basis functions (modified set of spherical harmonics), which are orthogonal on incomplete spheres. The number of basis functions for incomplete spheres is reduced and as a result the number of required loudspeakers is also reduced. These new basis functions need to be used in both the encoding and the decoding stage, which adds some complexity to this method compared to ALLRAD. Results presented in Zotter and Frank (2019) show that for hemispherical or incomplete spherical loudspeaker arrays, ALLRAD is the preferred method because it provides lower directional error, greater flexibility and it is simpler to implement compared to EPAD.

An N^th^-order conventional ambisonic decoder assumes $(N+1)^2$ uniformly distributed loudspeakers covering the sphere. For irregular distribution of loudspeakers, where the preferred methods are the ALLRAD and the EPAD, which in theory work for any loudspeaker arrangement it is still important to compute the equivalent ambisonic order for the specified loudspeaker set-up. A method to compute the equivalent ambisonic order for irregular loudspeaker set-up is presented in Zotter et al. (2012).

The design of the decoders can be either frequency-dependent or frequency-independent. Some earlier first-order decoding methods can incorporate frequency dependency by using shelf-filters or by using dual-band decoding matrices (one for the low and one for the high frequencies) together with phase-matched band-splitting filters (Heller et al. 2012). The goal of such decoders is to achieve the following: (1) Constant amplitude gain (important for low frequencies) and energy gain (important for high frequencies) across all angles; (2) Optimising the velocity localisation vector ${\textbf{r}}_{\text {V}}$, which is related to low-frequency localisation, and the energy localisation vector ${\textbf{r}}_{\text {E}}$, which is related to high-frequency localisation (${\textbf{r}}_{\text {V}}$ and ${\textbf{r}}_{\text {E}}$ have been defined by Gerzon (1992)). HOA methods such as EPAD and ALLRAD are not frequency-dependent and their main focus is the optimisation of the ${\textbf{r}}_{\text {E}}$. The performance of the decoders can be improved with the use of the max-${\textbf{r}}_{\text {E}}$ weights, which have been introduced by Daniel (2000). These are a set of weights that optimise the energy concentration in the expected direction by weighting each spherical harmonic component by an order-depended gain, which helps in preserving the total energy in the sweet spot (Favrot 2010) making max-${\textbf{r}}_{\text {E}}$ decoders best suited for high-frequency reproduction. However, recent work from Kuntz et al. (2023) indicates that max-${\textbf{r}}_{\text {E}}$ weights lead to higher sound-field reproduction errors under anechoic conditions when the receiver moves away from the centre of the loudspeaker array. The authors attribute the cause of this issue to the destructive interferences occurring between the signals generated by the loudspeakers. On the contrary other research (Frank et al. 2008; Zotter and Frank 2019) has shown that in non-anechoic environments, where room reverberation helps mitigate destructive interferences between the loudspeaker signals, max-${\textbf{r}}_{\text {E}}$ weights led to enhanced localisation at off-centre positions and minimised coloration fluctuations for moving sources.

In the literature it is suggested to create multi-band HOA decoders. For example, one can use a non-weighted decoder that focuses on amplitude preservation at low frequencies, and a decoder with max-${\textbf{r}}_{\text {E}}$ weights at high frequencies. A method to estimate the crossover frequency between the decoders is suggested in Favrot (2010). When the objective is to decode ambisonics recordings, one can use a separate decoder for each of the frequency ranges of the specific ambisonics microphone. According to McKenzie et al. (2021), the Zylia microphone delivers 2^nd^-order signals above 250 Hz and 3^rd^-order above 900 Hz. Based on these considerations, an ideal frequency dependent HOA decoder may consist of a 1^st^-order non-weighted decoder for frequencies up to 250 Hz, a 2^nd^-order decoder with max-${\textbf{r}}_{\text {E}}$ weights for frequencies between 250 Hz and 900 Hz, and a 3^rd^-order with max-${\textbf{r}}_{\text {E}}$ weights above 900 Hz. These frequency limits are not exact since, apart from the characteristic of the microphone array, they also depend on the choice of the encoding filters.

### Visual presentation of outdoor environments

The visual information in laboratory experiments can be presented via a screen, a projector or via a head mounted display (HMD). The visual information can be either recorded using a conventional camera, which records a rectangular image of the scene in front of the lens with a limited field of view, or a panoramic 360^∘^ camera, which records the whole environment surrounding the camera. It can also be generated using computer graphics. The advantage of using a conventional camera is that the video quality can be outstanding, but the subsequent information presentation in the lab can be either a rectangular 2D image or, in the best case, a 180^∘^ panorama. The 360^∘^ camera allows for 360^∘^ video playback via a HMD, where viewers can rotate their head around and have a much more immersive experience of the environment. The disadvantages are that the video quality is slightly inferior to that of a conventional camera and that it requires post-processing such as stitching. However, nowadays there are software packages that do all this automatically, requiring only minor refinements from the users. The 360^∘^ video is split into two categories, the monoscopic and the stereoscopic video. Monoscopic video means that during playback via the HMD, the same image is directed to both eyes by projecting a flat image to a sphere (360^∘^ panorama). As a result, depth perception is lost. Nowadays, there are cameras available on the market (in the upper price segments) that offer stereoscopic video, which resembles human vision. Although the stereoscopic video can provide a more immersive experience, capture and editing (stitching etc.) are more challenging, especially in dynamic scenes (Guillemaut et al. 2010). Badly produced 360^∘^ video can cause tremendous discomfort to the viewer, nausea and headaches. Computer generated graphics of real or imaginary environments have the advantage of providing an interactive experience to the user because they allow them to freely move inside the simulated environment using controllers and the depth information is superior to the recorded video. However, the environment is artificial compared to a 360^∘^ video recordings.

Multiple papers such as Yeo et al. (2020), Mostajeran et al. (2021), and Knaust et al. (2022) have looked into the effect of the different video reproduction methods in delivering visual information of urban and natural environments to viewers. Yeo et al. (2020) compared (a) 2D video viewed on a high-definition TV screen; (b) 360^∘^ video VR (360^∘^-VR) viewed via a HMD; and (c) interactive computer-generated VR (CG-VR), also viewed via a HMD and interacted with by using a hand-held controller, on their ability to deliver virtual nature for reducing boredom.^1^ The results showed that there was no significant difference in the reduction of boredom between the TV and the HMD conditions. However, a greater sense of presence was reported for the HMD conditions, with the computer-generated VR rated higher than the 360^∘^ recorded video. Mostajeran et al. (2021) investigated the effect of exposure to immersive 360^∘^ (monoscopic) videos and conventional 2D photo slideshows (also presented via the HMD) of forest and urban environments on mood, stress, physiological reactions, and cognition. The result showed that 2D photos were more effective in reducing physiological arousal compared to 360^∘^ videos, even though the 360^∘^ videos provided a stronger sense of presence. Knaust et al. (2022) looked into the exposure to virtual nature via 360^∘^ monoscopic video with HMD, via PC screen and an audio-only control condition (no video, just natural sounds) on skin conductance level, heart rate and perceived relaxation after participants have been experimentally stressed. The results showed that (a) the 360^∘^ was perceived as significantly more relaxing; (b) no significant difference was found between the 360^∘^ video and the PC screen regarding skin conductance; (c) there were no significant differences between the three conditions regarding the heart rate.

Generally, 360^∘^ video produces a high degree of presence in viewers. For video reproduction of urban outdoor environments, which are a central part in this work, where objects such as vehicles and humans move at different distances and speeds, using a monoscopic instead of a stereoscopic 360^∘^ video is the safer choice. It should be pointed out that the VR technology as well as the quality of the cameras are constantly improving. Thus, it is expected that conclusions drawn using current equipment and software might not necessarily be valid in the near future.

## Methodological overview

**Fig. 1:**
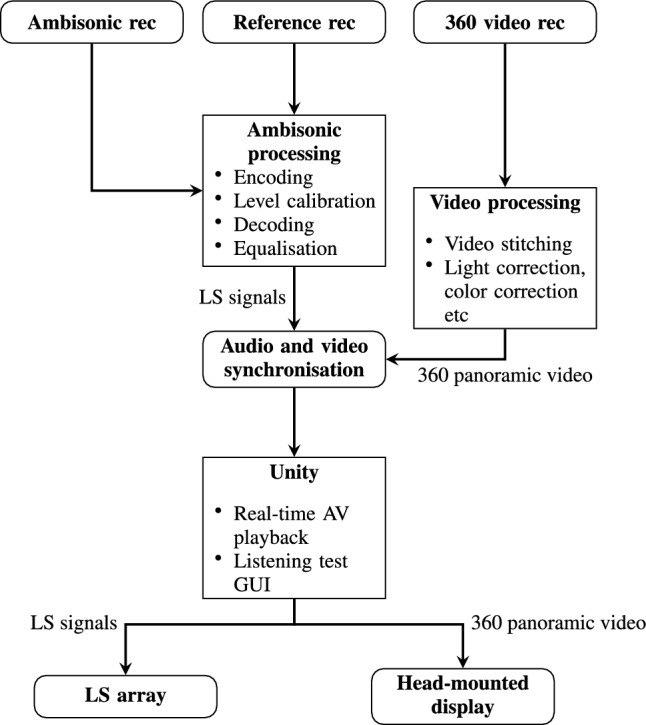
Block diagram of the audio-visual processing. LS = loudspeaker, AV = audio-visual, GUI = graphical user interface

The methodology workflow is illustrated in Fig. 1. First, audiovisual recordings are captured using an ambisonic microphone, a reference Class 1 measurement microphone, and a 360^∘^ camera (Sect. 6.1). The ambisonic recordings are then post-processed to produce the loudspeaker feeds (see Sects. 4 and 5), with the reference recordings being used for sound level calibration. The 360^∘^ video is stitched and undergoes color-and-light correction. Next, the pre-processed audio and video signals are time-synchronised and fed into the Unity game engine (Sect. 6.2). Finally, the multi-channel audio streams are sent to loudspeakers and the video is presented to a head-mounted display.

## Ambisonic recordings and equalisation

To perform sound field recordings within outdoor environments, the HOA microphone of the type Zylia ZM-1 (3^rd^-order) was selected. The self-noise of the microphone is relatively high, but in the outdoor soundscape recordings taken during this research, which were above 45 dB LAeq and also included background traffic noise, it was masked and hence imperceptible.

**Fig. 2:**
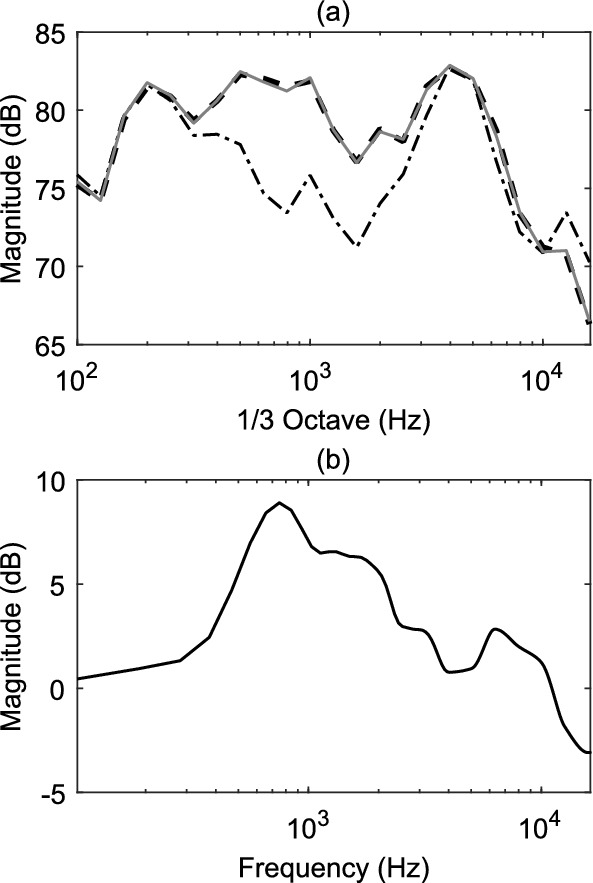
**a** Frequency response in 1/3 octave-bands of the reference omnidirectional microphone (dashed), Zylia 0^th^-order ambisonics channel (dashed-dotted) and its equalised version (solid); **b** The frequency response of the minimum phase filter used to equalise the Zylia recordings

The omnidirectional frequency response of the HOA microphone was measured in the laboratory. The 0^th^-order ambisonics channel (encoded with the Zylia Ambisonics Converter plug-in v.1.7.0) has information about the pressure field at the origin, thus, it is equivalent to an omnidirectional microphone at the origin. The frequency response of the Zylia was measured inside the reverberation chamber at the authors’ institution Empa, using pink noise. The reason for measuring inside the diffuse field of the reverberation chamber was to feed all the capsules with almost identical signals and to derive a diffuse field equalisation. The same measurements were repeated using the calibrated measurement microphone Norsonic Type 1209/15268 as a reference. In Fig. 2a the frequency response in 1/3 octave-bands of the 0^th^-order ambisonic channel of Zylia is plotted (dotted line) against the reference microphone (dashed line). In the frequency range around 1 kHz the two curves deviate by more than 5 dB. The reason for this difference is due to the ambisonics encoding. However, due to the use of the Zylia Ambisonics Converter plug-in v.1.7.0, pinpointing the precise cause remains inconclusive (potentially attributed to factors such as the frequency response of the radial filters). In order to correct this difference, a minimum-phase FIR filter was computed based on the 1/3 octave-band spectrum difference of the Zylia 0^th^-order ambisonic channel and the reference. The filter is plotted in Fig. 2b and the corrected 1/3 octave-band spectrum of the Zylia 0^th^-order ambisonic channel is plotted in Fig. 2a with a solid line. This equalisation filter was applied to all the channels of the B-format.

For the selected HOA microphone, correction gains of more than 5 dB were required to equalise its frequency response with a substantial boost of the important frequency range from around 500 Hz to 2 kHz. This shows the importance of testing and correcting the frequency response of commercial HOA microphones before use in scientific or noise-related context.

## Ambisonic decoder variants

### Sound reproduction room: AuraLab

**Fig. 3:**
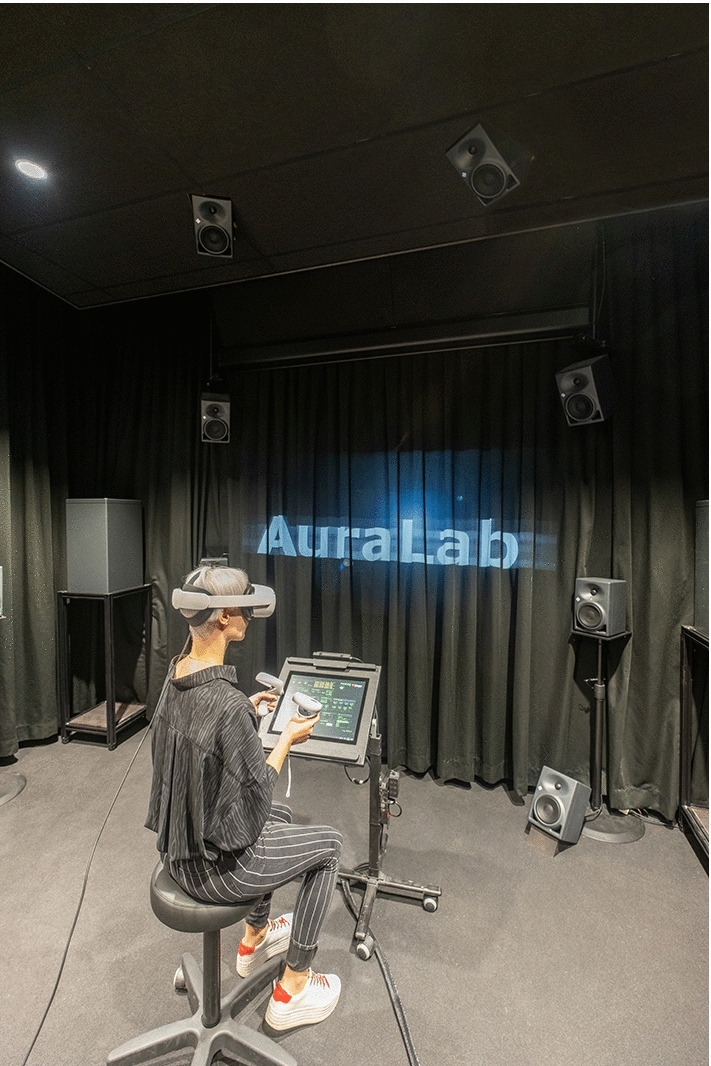
AuraLab listening test facility at the Laboratory for Acoustics/Noise Control at Empa showing the spherical loudspeaker array surrounding a listener wearing a head-mounted display in a highly controlled acoustic environment

**Fig. 4:**
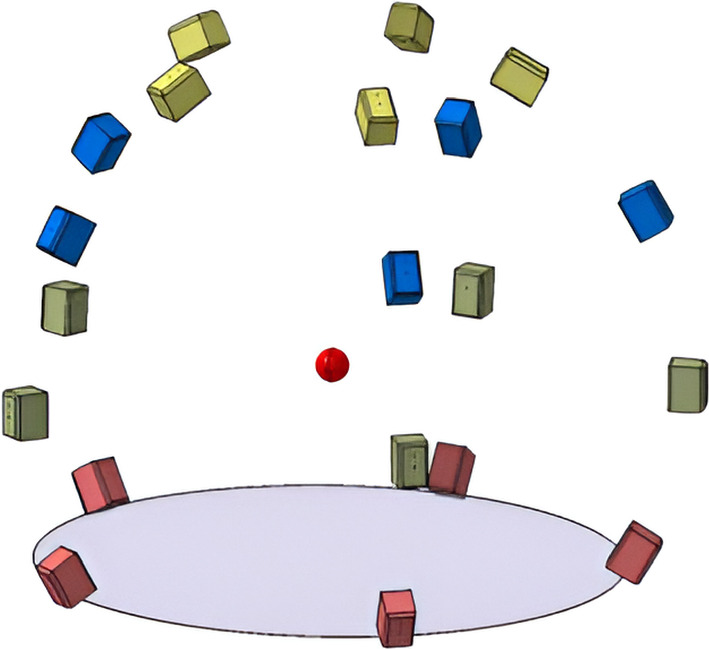
Graphical representation of the satellite loudspeaker configuration in AuraLab around the listening position (red dot). Loudspeakers sharing the same color indicate they are positioned at the same elevation and height above ground (ellipse) (Color figure online)

The sound reproduction takes place inside the listening test facility *AuraLab* (Auralisation Laboratory; Fig. 3) at Empa in Switzerland. The listening room has a volume of 70 m^3^ and is acoustically-treated. It contains 20 satellite loudspeakers (Neumann KH 120 A) and four subwoofers (Neumann KH 805). The satellite speakers are placed on a sphere around the listener, with a listening distance of 2 m at four elevation angles ($-28^{\circ }$,$0^{\circ }$,$30^{\circ }$,$60^{\circ }$) and at each elevation plane there are five loudspeakers at $36^{\circ }$, $108^{\circ }$, $180^{\circ }$, $252^{\circ }$ and $324^{\circ }$ (see Fig. 4 ). The speakers below the listening plane were added particularly for this study to test the benefit of different ambisonic decodings. Four distributed subwoofers are installed with a crossover frequency of 100 Hz. All speakers are connected to two 16-channel programmable digital signal processors (Xilica Neutrino A 0816, 48 kHz sampling rate) which receive digital multichannel audio streams from a computer over Ethernet using the DANTE protocol.

**Fig. 5:**
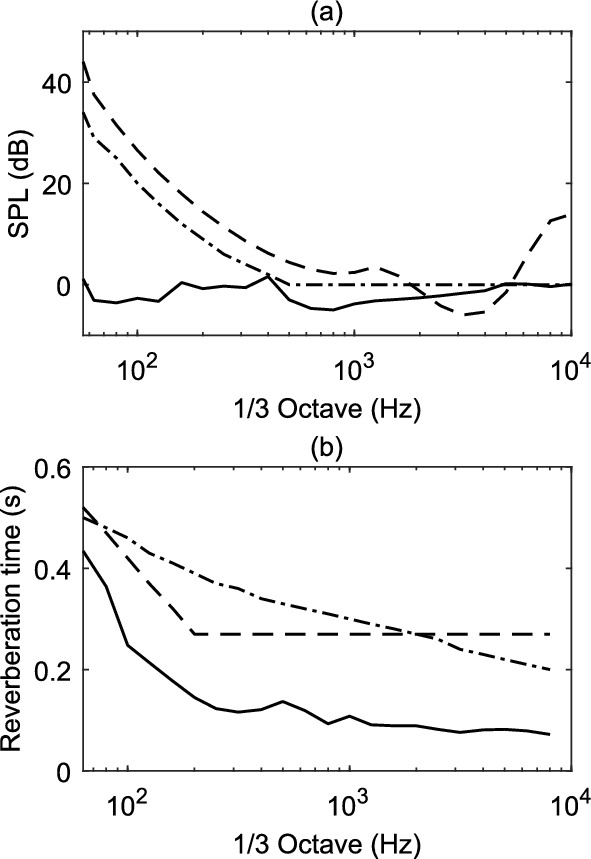
**a** Measured background noise inside AuraLab (solid) plotted against the hearing threshold from ISO 226:2003 (dashed) (International Organization of Standardization 2003) and the GK0 upper limit (dashed-dotted) (Institut für Rundfunktechnik 1995); **b** Measured reverberation time inside AuraLab (solid) plotted against upper Dolby limit (dashed) (Dolby Laboratories Inc 2005) and upper THX limit (dashed-dotted) (THX Lucasfilm Ltd 1999)

In Fig. 5, the background noise (below 7 dB(A)) and the reverberation time (0.11 s in mid-frequencies) measured inside AuraLab are shown and compared against professional listening room standards and recommendations (IRT Institut für Rundfunktechnik 1995, Dolby Dolby Laboratories Inc 2005 and THX THX Lucasfilm Ltd 1999), and the hearing threshold curve ISO 226:2003 (International Organization of Standardization 2003). As shown, the room and the system fulfills the highest requirements on professional listening rooms.

The air temperature and CO2 levels inside AuraLab are constantly monitored using the EmpAIR system (EmpAIR 2021) and the data are sent to the PC in the control room. The control room hosts an audio-visual supervision system, a talkback microphone, a screen duplicating the HMD and the computer with a screen to steer the experiment.

### Tested decoders

A listening experiment was conducted to evaluate the four decoders listed below, with participants rating the relative (perceived) realism of outdoor soundscapes characterised by relatively low sound pressure levels (SPL). The studied decoders are the following:

1. 2D FOA max-${\textbf{r}}_{\text {E}}$ decoder using only the $L = 5$ speakers at $0^{\circ }$ elevation, which will be referred to as $FOA_{2D}$
2. 3^rd^-order HOA max-${\textbf{r}}_{\text {E}}$ ALLRAD decoder for the hemispherical loudspeaker setup ($L = 15$ speakers layout), which will be referred to as $HOA_\text {hemi}$
3. 3^rd^-order HOA max-${\textbf{r}}_{\text {E}}$ ALLRAD decoder making use also of the ground speakers at $-28^{\circ }$ elevation ($L = 20$ speakers layout), which will be referred to as $HOA_\text {full}$
4. Dual-band decoder in which for frequencies up to 400 Hz a 1^st^-order unweighted ALLRAD decoder and for higher frequencies a 3^rd^-order max-${\textbf{r}}_{\text {E}}$ ALLRAD decoder is used ($L = 20$ speakers layout), which will be referred to as $HOA_\text {full,dualband}$

All HOA encoding was done with the Zylia Ambisonics Converter plug-in v.1.7.0, using full 3D normalisation (N3D) and ambisonic channel number (ACN) ordering format. For FOA, it is more common to use the FuMa format. If the user chooses the FuMa channel ordering from the Zylia Ambisonics Converter software, one might expect that FuMa normalisation is also applied, which is referred to in the literature as maxN normalisation. However, the Zylia software gives only the option to apply the N3D or the SN3D normalisation. To convert from ACN channels ${\textbf{C}}$ with N3D normalisation to the FuMa format, the following transformation is made:

2 $$\begin{aligned}{}[W,Y,Z,X]= {\textbf{C}} \ \text {diag}\left( {\textbf{w}} \right) \end{aligned}$$

where $${\textbf{C}} = [C_0,C_1,C_2,C_3]$$ and $${\textbf{w}}= \left[ \frac{1}{\sqrt{2}},\frac{1}{\sqrt{3}},\frac{1}{\sqrt{3}},\frac{1}{\sqrt{3}}\right]$$

Equation 3 is used for the $FOA_{2D}$ decoder.

3 $$\begin{aligned} {\textbf{s}}_\text {n} = K_{0} W + K_{1}[X \cos (\varphi _\text {n}) + Y \sin (\varphi _\text {n})] \end{aligned}$$

where ${\textbf{s}}_\text {n}$ are the loudspeaker signals for the $n^{\text {th}}$ loudspeaker located at azimuth angle $\varphi _\text {n}$, *W*, *Y*, *X* are the B-format channels and the weights $K_{0} = K_{1} = \sqrt{2}/L$, where $L=5$ is the number of speakers. The omnidirectional channel *W* is defined as $W=p/\sqrt{2}$, where *p* is sound pressure. The reason for setting the weights $K_{0} = K_{1}$ is to yield a max-${\textbf{r}}_{\text {E}}$ decoder, which works well for frequencies 400 Hz and above and also provides the best reproduction if no frequency-dependent FOA decoding is applied (Benjamin et al. 2006).

The HOA ALLRAD decoding matrix was computed using the following equation:

4 $$\begin{aligned} {\textbf{D}}_{\text {allrad}} = \frac{4\pi }{J} \textbf{GY}_\text {N}^{T} \end{aligned}$$

where $J = 240$ is the number of virtual loudspeakers in the optimal spherical virtual loudspeaker layout (Hardin and Sloane 1996), ${\textbf{G}}$ is a $L \times J$ matrix that contains the gains of all real loudspeakers *L* for creating all *J* virtual loudspeakers and ${\textbf{Y}}_\text {N}$ is a $J \times N$ matrix ($N = 3$, the ambisonic order) that contains the real-valued orthonormalised spherical harmonics for the positions of the *J* virtual loudspeaker up to order $N = 3$. The max-${\textbf{r}}_{\text {E}}$ weight for the $n^{th}$-order is computed using the formula below, taken from Zotter and Frank (2012):

5 $$\begin{aligned} a_\text {n}=P_\text {n}\left( \cos \left( \frac{137.9^{\circ }}{N+1.51}\right) \right) \end{aligned}$$

where $P_\text {n}$ are the Legendre polynomials. For $N=3$ the weights $a_0$ to $a_3$ are [1, 0.861, 0.612, 0.304]. The max-${\textbf{r}}_{\text {E}}$ weights are integrated in equation 4 by:

6 $$\begin{aligned} {\textbf{D}}_{\text {allrad}} = \frac{4\pi }{J} \textbf{GY}_\text {N}^{T} \text {diag}({\textbf{a}}) \end{aligned}$$

where $${\textbf{a}}= [a_0, a_1,a_1,a_1,..., a_n]^T$$.

The HOA decoding is implemented using the ambisonics Matlab library developed by Politis (2016). In all three HOA decoders, two imaginary speakers are inserted at $\pm 90^{\circ }$ elevation to fill in the gaps in the loudspeaker layout as recommended in Zotter and Frank (2019) and Heller and Benjamin (2014). The signal of the imaginary speaker is disposed as suggested in Zotter and Frank (2019). Although, whether the signals from the imaginary speakers should be decorrelated and then mixed into the nearby speaker remains an open question (Heller and Benjamin 2014).

The minimum amount of loudspeakers required for 3^rd^-order ambisonic reproduction is $L = 16$ speakers. In hemispherical loudspeaker layouts, such as the one used with decoder $HOA_\text {hemi}$, the bottom half can be omitted when using the ALLRAD or the EPAD with the modified basis functions, meaning that this layout could produce up to 4^th^-order ambisonics on the top hemisphere (Zotter and Frank 2019). However, since the Zylia ZM-1 can provide up to 3^rd^ ambisonics signals, there is no benefit in that. Some psychoacoustic evaluations in 2D HOA in the literature (an overview of this research can be found in Zotter et al. (2010) and Politis (2016)) have shown that artifacts such as comb-filtering and spectral impairment occur if the number of the loudspeakers is larger than the one required (hence more interference) from the ambisonic order. Therefore, one could claim that using 15 loudspeakers for producing a 3^rd^-order sound field in the hemisphere might be a lot. However, the amplitude, energy, velocity vector and energy vector plots showed that the results were best when all loudspeaker positions where used.

**Fig. 6:**
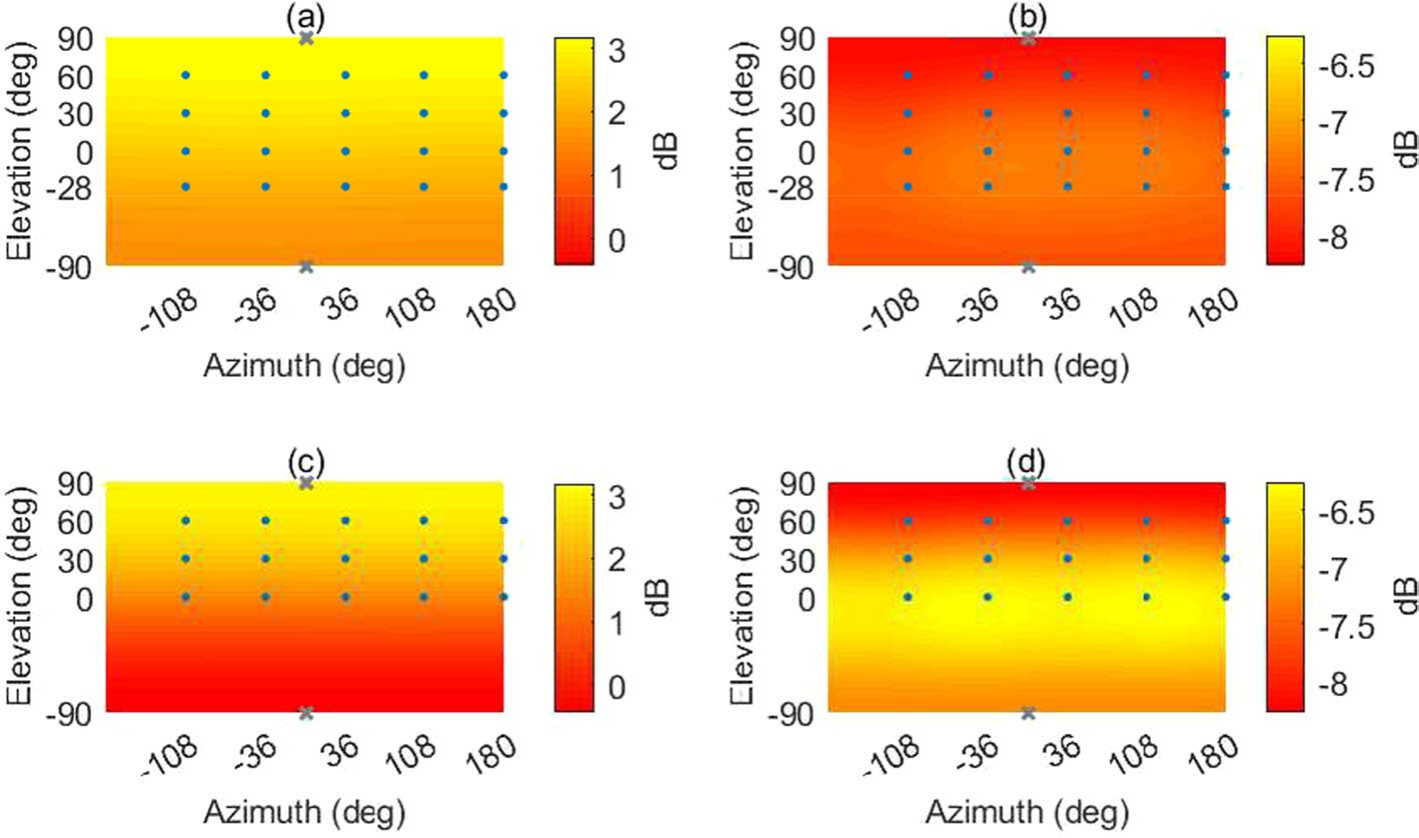
Amplitude **a** and energy **b** distribution plots of $HOA_\text {full}$ and amplitude **c** and energy **d** distribution plots of $HOA_\text {hemi}$. The dots indicate the locations of the speakers and the crosses the locations of the imaginary loudspeakers

The $HOA_\text {hemi}$ and $HOA_\text {full}$ make use of the max-${\textbf{r}}_{\text {E}}$ for the whole frequency range (Zotter and Frank 2012). In earlier studies, there were no speakers below the listening plane in Auralab. Analysis of the amplitude and energy distributions of the $HOA_\text {hemi}$ and the $HOA_\text {full}$ decoder in Fig. 6 shows that the reproduction could potentially be improved by creating more even sound distributions (also with respect to the horizontal plane). Another reason for adding the ground speakers at $-28^{\circ }$ is that in some recording locations some noise sources were below the ear height, but with the hemispherical array, on some occasions, they were reproduced above the ear height.

In $HOA_\text {full,dualband}$ the max-${\textbf{r}}_{\text {E}}$ weights were only applied in the mid-high frequency decoder. The cross-over between the two decoders is done using 4^th^-order Linkwitz-Riley filters.

### Decoder equalisation

Once a person sits in the centre of a loudspeaker array, their head and torso affect the sound field by shielding and reflecting the sound waves. The shielding effect by the head becomes more dominant at higher frequencies since the wavelengths become smaller compared to the size of the head. To remove any direction-independent effects of the ambisonic reproduction, diffuse-field equalisation is applied. More specifically, diffuse-field equalisation shelf-filters are applied on all four decoders that are derived based on a virtual loudspeaker simulation and HRTFs (*H*). The computation of the equalisation shelf-filter is described in this subsection. It is important to highlight that an individual equalisation shelf-filter was calculated for each decoder. Moreover, this decoder equalisation method does not take into account the room effect into the perceived sound field. The room effect is separately considered by treating the room with broadband sound absorbers on all room surfaces (see information regarding the reproduction room in Sect. 5.1).

**Fig. 7:**
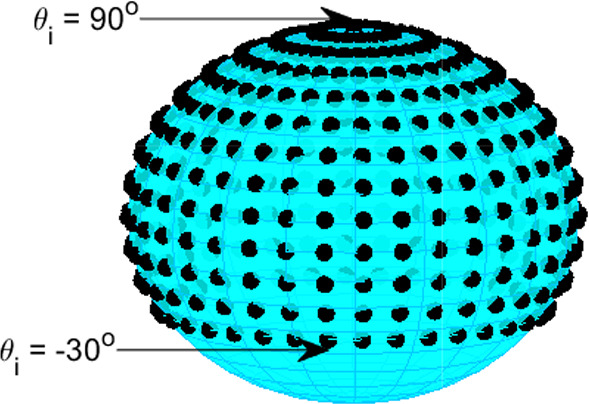
HRTF locations used for diffuse field average computation

**Fig. 8:**
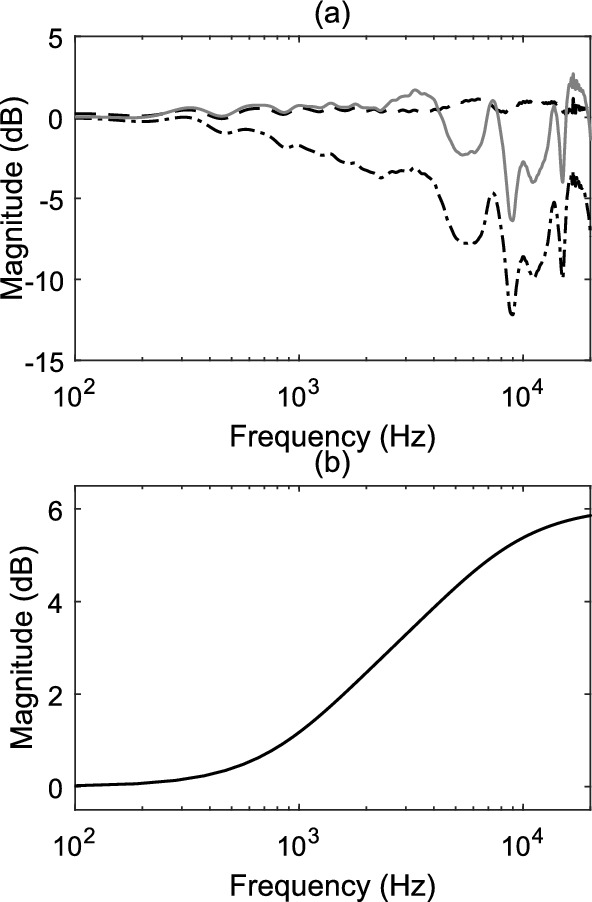
**a** Diffuse-field energetic average of the measured HRTFs (dashed), ambisonically rendered HRTFs using the $HOA_\text {full}$ decoder (dashed-dotted) and its equalised version (solid); **b** The shelf-filter used to equalise the loudspeaker signals computed with the $HOA_\text {full}$ decoder

Firstly, artificial ambisonic channel signals are created for both 1^st^-order FuMa channel ordering and weighting (for the 2D FOA decoder), and 3^rd^-order ACN channel ordering and N3D weighting (for the HOA decoders) by encoding *M* virtual sources *i* at coordinates on the surrounding sphere. The ambisonics signals are computed for azimuth angles $\phi _\text {i}$ starting from $0^{\circ }$ to $360^{\circ }$ and for elevation angles $\theta _\text {i}$ from $-30^{\circ }$ to $90^{\circ }$ (see Fig. 7). Both the azimuth and elevation increments are set to $\Delta \phi$ = $\Delta \theta$ = $10^{\circ }$. In total, $M=468$ positions around the sphere are encoded (the encoded signal is a Kronecker delta $\delta$). The encoding of the Kronecker delta at the $i^{th}$ position was implemented by multiplying $\delta$ with the real orthonormalised spherical harmonic value of this position up to order *N*:

7 $$\begin{aligned} {\textbf{x}}_{i} = \mathbf {\delta Y}_\text {N}^{T} \text {diag}({\textbf{a}}) \end{aligned}$$

where $\mathbf {Y_{N}}$ is a $1 \times (N+1)^2)$ vector with the real orthonormalised spherical harmonic values of this position up to order *N*. The 1^st^-order ambisonics signals for each position *i* are decoded for the horizontal 2D loudspeaker layout using the $FOA_{2D}$ decoder, the 3^rd^-order signals are decoded for the hemispherical loudspeaker layout using the $HOA_\text {hemi}$ and for the layout with the ground speakers at $-28^{\circ }$ elevation using the $HOA_\text {full}$ and the $HOA_\text {full,dualband}$. Secondly, each of the loudspeaker signals *s* for each position *i* is convolved with the corresponding head related impulse response (*h*) of the SADIE HRTF database (Kearney and Doyle 2015) (KEMAR dummy head). The measured angles of the HRTF database do not perfectly match the angle of all loudspeakers. They are on average $1.5^{\circ }$ off, thus, the virtual representation of the layout is not fully correct. Thirdly, the diffuse-field energetic average level of the ambisonically rendered HRTFs ($L_{\text {DF,amb}}$) is computed with the following equation:

8 $$\begin{aligned} L_{\text {DF,amb}} = 10 \log _{10} \left( \frac{1}{M} \sum _{i = 1}^{M}\left| {\mathcal {F}} \left( \sum _{n=1}^{L}\left( s_\text {n,i}*h( \varphi _\text {n}, \theta _\text {n}) \right) \right) \right| ^{2} \Omega _\text {i} \right) \end{aligned}$$

with *L* being the number of speakers in the layout, $s_\text {n,i}$ the $n^{\text {th}}$ loudspeaker (with polar coordinates ($\phi _\text {n}$,$\theta _\text {n}$)) signal for the $i^{\text {th}}$ virtual source position and $\Omega _\text {i}$ the solid angle weight ($\Omega =\cos (\theta )\Delta \theta \Delta \varphi$) for the corresponding $i^{\text {th}}$ virtual source position. The HRTFs are multiplied with the corresponding solid angle weight in order to ensure no specific direction will be over-represented in the average. The diffuse-field energetic average of the measured HRTFs at the *M* coordinates is computed with equation 9. From the selected $M=468$ positions in the above mentioned analysis, 35 HRTFs were missing from the SADIE HRTF database and they are excluded from the analysis. Finally, a second-order infinite impulse response (IIR) shelf-filter is designed (using Matlab’s *designShelvingEQ* function) for each decoder to equalise it and bring its diffuse-field spectrum closer to the measured spectrum as shown in Fig. 8. Using generic HRTFs in this case is not anticipated to result in any notable drawbacks, as the equalisation filter functions as a shelf-filter. As shown in Fig. 8, its objective is to align closely the diffuse-field spectrum computed using ambisonically rendered HRTFs to the one computed using measured HRTFs, rather than to correct for all the peaks and valleys of the diffuse-field spectrum, which differ among individuals. The approach followed here is very similar to the one from McKenzie et al. (2018), which was developed for binaural reproduction of ambisonics. The main difference is that instead of using a shelf-filter they created an equalisation filter based on the inverse diffuse-field response in order to flatten it.

The derived equalisation filter shown in Fig. 8 for the HOA decoder realises an amplification of the high frequencies by 6 dB. In the relevant frequency range of 2-3 kHz, the amplification amounts to about 3 dB. These magnitudes show the relevance of considering the effect of the listener on the sound field in HOA for an accurate sound reproduction.

9 $$\begin{aligned} L_{\text {DF,meas}} = 10 \log _{10} \left( \frac{1}{M} \sum _{i =1}^{M} \left| H(\varphi _\text {i},\theta _\text {i}) \right| ^{2} \Omega _\text {i} \right) \end{aligned}$$

### Perceptual decoder evaluation

#### Test signals

**Fig. 9:**
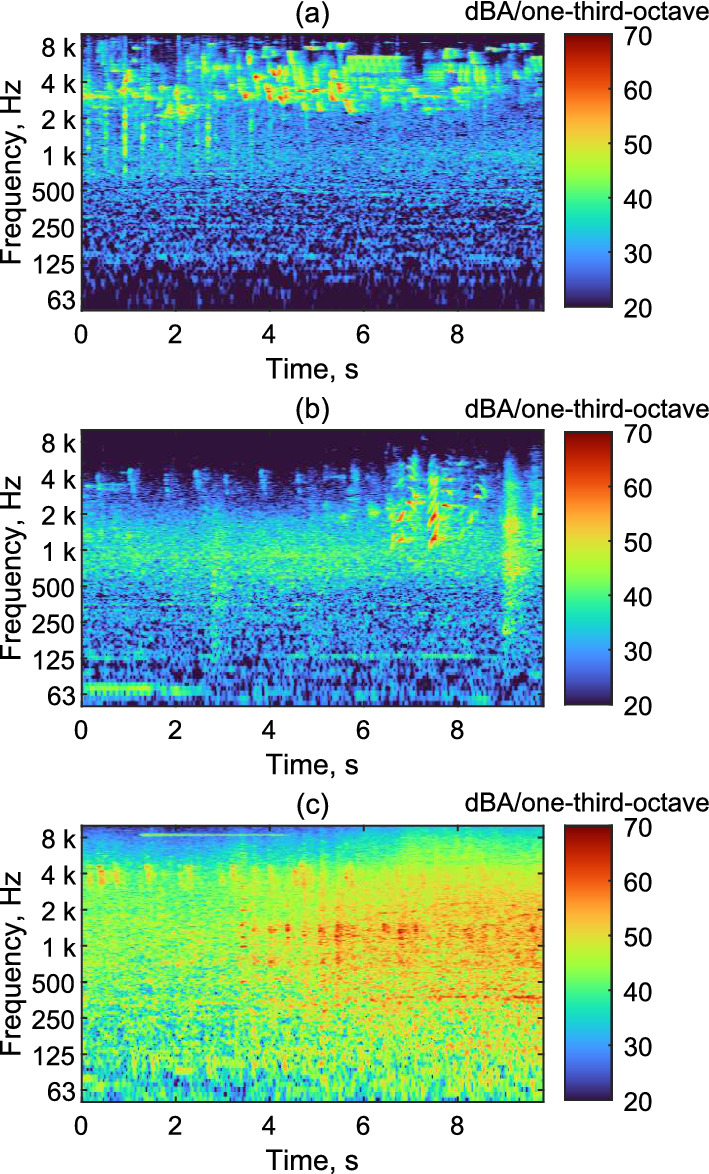
Spectrograms (1/3 octave-band spectra) of the three A-weighted outdoor soundscapes recordings **a** soundscape dominated by sound of nature; **b** Soundscape dominated by human voices; **c** Soundscape dominated by road traffic noise

Three different outdoor soundscape recordings of 9 s duration were chosen for the psychoacoustic decoder evaluation experiment. The first recording was dominated by sound of nature (bird songs, leaves rustling due to mild wind), the second by human voices (mainly children playing at distance), and the third by road traffic noise. Background road traffic noise from a street at a distance of 40 m was also present in the first two recordings, but did not dominate the soundscapes. The recordings were taken using the methodology presented in Sect. 6.1. The spectrograms of the A-weighted outdoor soundscape recordings are plotted in Fig. 9.

#### Listening test methodology

**Fig. 10:**
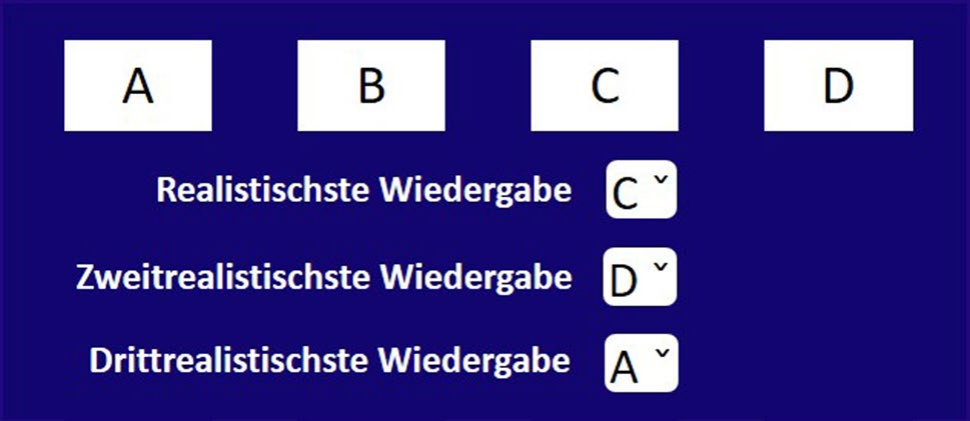
Experimental GUI

The goal of this listening experiment was to assess the relative (perceived) realism of each of the four decoders ($FOA_{2D}$, $HOA_\text {hemi}$, $HOA_\text {full}$, $HOA_\text {full,dualband}$). Perceived realism can be interpreted as a perceptual quality that indicates how close the reproduced sound-field feels like natural listening to the participants. As we lacked a ground truth condition, one should interpret this as the plausibility rating within an internal frame. The test consisted of four trials, the first one being the training trial. At each trial, participants could listen to one of the three soundscape recordings, decoded with the four different decoders. Their task was to rank the differently decoded soundscapes as 1^st^, 2^nd^ and 3^rd^ most realistic (the unranked sample was considered the least realistic). We chose a forced-choice ranking of relative realism (without the option "equally realistic") over a direct rating of absolute realism, in order to be able to detect very subtle possible differences between the decoders. Participants could playback the audio signals as many times as they wanted before they performed the ranking, but they had to listen at least once to each signal before they were allowed to do the ranking. The training trial was the same for all subjects and was excluded from analysis. The order of the other three trials as well as the positions of the four decoders were balanced over all participants. The playback and ranking was implemented using the GUI (written in German language) shown in Fig. 10. The participants received both verbal and written instructions. A convenience sample of thirteen volunteers, which included experts from the field of acoustics was tested (mean age 35.1±10.7 years; ten male, three female). The average duration of the test was 7 min 40 sec.

#### Results and discussion

**Fig. 11:**
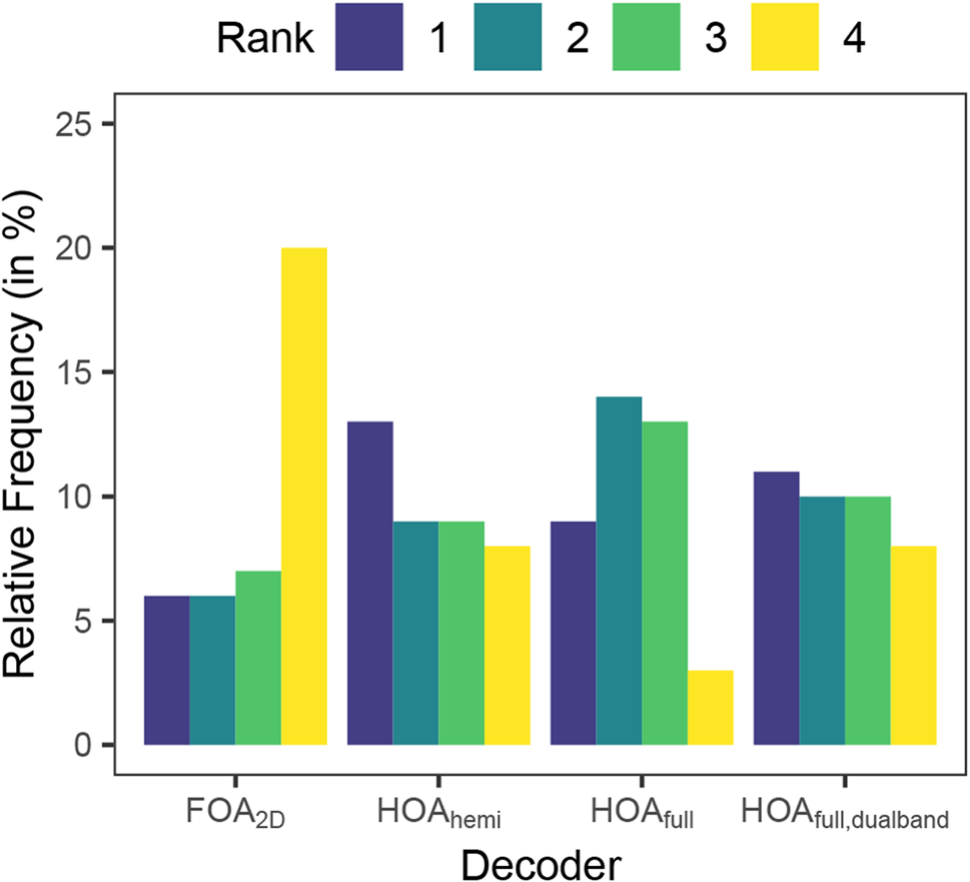
Relative frequencies of the realism ratings of the different decoders $FOA_{2D}$, $HOA_\text {hemi}$, $HOA_\text {full}$, $HOA_\text {full,dualband}$ per rank (from 1 most realistic to 4 least realistic)

Figure 11 visualises that the recordings decoded with $HOA_\text {full,dualband}$ were selected most often as most realistic and least often as least realistic. However, there was no significant difference between the three HOA decoders. In contrast, the recordings decoded with $FOA_{2D}$ were selected least often as most realistic and most often as least realistic. Pairwise Wilcoxon Rank Sum Tests confirmed that the $FOA_{2D}$ decoder was rated significantly less realistic than $HOA_\text {full}$ (*p* = 0.026), with $HOA_\text {hemi}$ and $HOA_\text {full,dualband}$ narrowly missing the significance level after false discovery rate (FDR) correction for multiple comparisons (*p* = 0.057 and *p* = 0.125, respectively).

**Table 1:** Median and mean rankings (± *SD*) for all decoders and soundscape (nature, traffic, voices). Larger values indicate lower perceived realism

	**Nature**			**Traffic**			**Voices**			**Total**		
	Median	Mean	SD	Median	Mean	SD	Median	Mean	SD	Median	Mean	SD
$FOA_{2D}$	3	3.31	0.75	4	3.31	1.18	2	2.54	1.33	4	3.05	1.15
$HOA_\text {hemi}$	2	2.38	0.87	2	1.92	0.95	3	2.85	1.34	2	2.38	1.11
$HOA_\text {full}$	2	1.92	0.95	2	2.46	0.88	2	2.38	0.87	2	2.26	0.91
$HOA_\text {full,dualband}$	2	2.38	1.45	2	2.31	1.11	2	2.23	0.93	2	2.31	1.15

Considering the three soundscapes individually, Table 1 shows that for the soundscape dominated by human voices, all decoders were ranked similarly, even though the FOA decoder is a 2D decoder. This could be because for voices it is very natural and most common in everyday life to be perceived from ear height. In contrast, the 2D FOA decoder performed worse for the traffic and nature soundscape. This could be due to the fact that these soundscapes were more complex and dominated by sound produced also above ear height (bird song and rustling leaves). The $HOA_\text {hemi}$, which employs a hemispherical loudspeaker setup, was rated as more realistic in the traffic soundscape. One might expect the $HOA_\text {full}$ and the $HOA_\text {full,dualband}$ to perform better, as they use a loudspeaker array that includes speakers below the ear height, where the main noise sources of the cars are located (engine and tire noise). However, it is worth noting that the microphone used in the recordings was positioned approximately 50 m away from the road at a height of 1.2 m (the height of a seated person). Consequently, sound sources like tire noise and engine noise would only deviate by approximately 1.2^∘^ below ear height. In general, all *HOA* decoders performed very similarly but clearly better than the $FOA_{2D}$. However, 2D FOA decoders should still be considered for soundscape reproduction in cases where a HOA system is not available for the following reasons: (1) They are much simpler to implement compared to HOA decoders; (2) The costs of the reproduction system required for FOA are lower; (3) HOA reproduction requires recordings taken with HOA microphones, which have high self noise due to their capsules and the encoding filter. So for very quiet soundscapes, HOA recording can be problematic and FOA could be a better option.

Since the physically most accurate decoder $HOA_\text {full,dualband}$ was selected most often as most realistic and least often as least realistic (even though the difference with the other HOA decoders was not significant), this decoder was chosen for the current VR setup.

#### Limitations and future tests

The equivalent ambisonics order for the hemispherical loudspeaker set-up used with the $HOA_{hemi}$ and the loudspeaker set-up with the ground speakers used with the $HOA_{full}$ and $HOA_{full,duallband}$ is 3.3 and 2.7, respectively. Even though the loudspeaker layouts are capable of reproducing 3^rd^-order ambisonics, the used loudspeaker distribution is sub-optimal. This is because the loudspeaker density is lower in the horizontal plane ($0^{\circ }$) and higher towards the poles. It would be interesting in future work to explore whether the ambisonic recordings of outdoor soundscapes are reproduced better in sub-optimal loudspeaker layouts using parametric reproduction methods such as higher-order directional audio coding (DirAC) (Politis et al. 2015).

The outdoor soundscapes were presented to the participants in the absence of visual cues. In future studies, it would be interesting to replicate the experiment with visual information included and assess how this variable influences the perceived realism of various ambisonic decoders.

## 360^∘^ audio-visual recording and playback

### Audio and video recordings

**Fig. 12:**
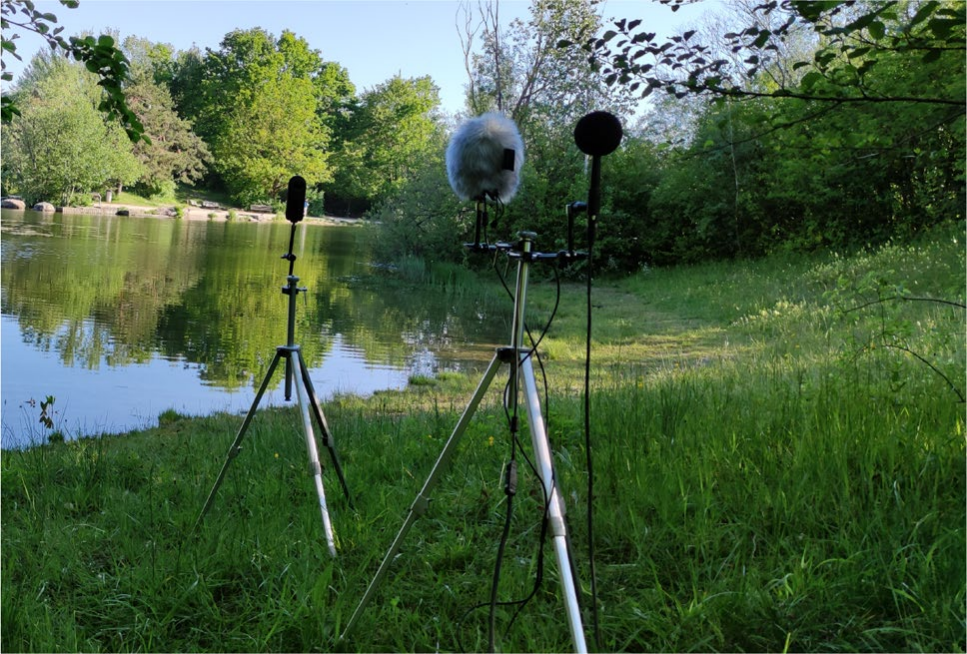
Field measurement setup with the reference sound pressure microphone with a foam windscreen (back), the HOA microphone with a fur windscreen (centre) and the 360^∘^ camera (front)

The used measurement setup for field data collection is shown in Fig. 12. The monoscopic 360^∘^ camera Kandao Qoocam was used for the video recordings. The Zylia microphone was placed at 1 m distance from the QooCam. In addition, a reference microphone (B&K 4006 with diffuse-field grid) was placed 30 cm from the Zylia microphone. The latter was connected to a laptop with Core i7 $8^{th}$ Gen with Windows 10 operation system via a 20 m USB 3.0 active extension cable. The reference microphone was connected via a 20 m XLR cable to an NTi Audio XL2-TA (Class-1 sound level meter), which recorded both SPLs and the audio signal. The height of the QooCam, the Zylia and the reference mic was set at 1.20 m, which is equivalent to the eye height of a seated person. The ambisonics recordings were taken at a sample rate of 48 kHz and the videos at 8K resolution and 30 FPS. The sound level meter stored SPLs every 1 s and recorded the audio signal at 48 kHz. At a close distance to the measurement location, the Rotronic A1 HYGROMER hydrometer and the Windmaster 2 wind speed meter were fixed in order to monitor the air temperature, relative humidity and wind speed. Windscreens were always placed on both mics (the Zylia original windscreen was used for the Zylia mic). Before each recording, the reference microphone was calibrated. The 1 kHz calibration tone was recorded in order to use it in post-processing to calibrate the reference recording, which was then used to calibrate the ambisonic recording.

### VR environment

The VR environment including the experimental GUI was implemented within the Unity game engine (version 2020.3.29f1). The video playback is done using the VideoPlayer class of Unity, via the HMD of type Oculus Quest 2. The Oculus Integration SDK v31 was used in order to enable hand tracking, which allows the participant to answer experimental questions on a virtual GUI using their own hands without the need of controllers. The audio playback is carried out using the BASS audio library (https://www.un4seen.com/). More specifically, the *BASS*.*dll* and *BASSASIO*.*dll* are imported into the Unity project. The $HOA_\text {full,dualband}$ is used for the reproduction (see Sect. 5.4). All decoding and equalisation is taking place in the pre-processing stage as shown in Fig. 1.

### Application

The methodology presented in this study was shown to be suited for application in psychophysical experiments on the effects of road traffic noise and green spaces on physiological and psychological outcomes (Kawai et al. 2023). While originally designed for these specific investigations, the methodology serves as a valuable blueprint for future VR and soundscape studies exploring the combined influence of exposure to different aural and visual stimuli on human perception, psychology and physiology.

## Conclusion

This paper presents a methodology designed to capture, post-process, and replicate audio-visual data of outdoor environments for VR experiments. The focus is to achieve accurate (physically correct) spatial sound reproduction within the VR environment, accomplished through the use of higher-order ambisonics along with microphone recordings and ambisonic decoder calibration and equalisation. The methodologies for equalising the ambisonic microphone recordings and the ambisonic decoders are presented. Analysis results emphasize the importance of considering both factors for achieving accurate ambisonic reproduction. Listening experiments were conducted to evaluate the relative (perceived) realism of reproduced outdoor soundscapes from ambisonic recordings decoded with four different decoders. The findings indicate that the performance of the 3D HOA decoders surpass that of the 2D FOA decoder. 3D HOA is thus recommended for spatial reproduction of outdoor urban soundscapes in the laboratory.

## Data Availability

Data will be made available upon request.

## Footnotes

1 "an unpleasant, transient affective state in which an individual feels a pervasive lack of interest"
